# Association between breastfeeding and intelligence, educational attainment, and income at 30 years of age: a prospective birth cohort study from Brazil

**DOI:** 10.1016/S2214-109X(15)70002-1

**Published:** 2015-04

**Authors:** Cesar G Victora, Bernardo Lessa Horta, Christian Loret de Mola, Luciana Quevedo, Ricardo Tavares Pinheiro, Denise P Gigante, Helen Gonçalves, Fernando C Barros

**Affiliations:** a Postgraduate Programme in Epidemiology, Universidade Federal de Pelotas, Brazil, Pelotas, Rio Grande do Sul, Brazil; b Postgraduate Programme in Health and Behavior, Universidade Católica de Pelotas, Pelotas, Rio Grande do Sul, Brazil

## Abstract

**Background:**

Breastfeeding has clear short-term benefits, but its long-term consequences on human capital are yet to be established. We aimed to assess whether breastfeeding duration was associated with intelligence quotient (IQ), years of schooling, and income at the age of 30 years, in a setting where no strong social patterning of breastfeeding exists.

**Methods:**

A prospective, population-based birth cohort study of neonates was launched in 1982 in Pelotas, Brazil. Information about breastfeeding was recorded in early childhood. At 30 years of age, we studied the IQ (Wechsler Adult Intelligence Scale, 3rd version), educational attainment, and income of the participants. For the analyses, we used multiple linear regression with adjustment for ten confounding variables and the G-formula.

**Findings:**

From June 4, 2012, to Feb 28, 2013, of the 5914 neonates enrolled, information about IQ and breastfeeding duration was available for 3493 participants. In the crude and adjusted analyses, the durations of total breastfeeding and predominant breastfeeding (breastfeeding as the main form of nutrition with some other foods) were positively associated with IQ, educational attainment, and income. We identified dose-response associations with breastfeeding duration for IQ and educational attainment. In the confounder-adjusted analysis, participants who were breastfed for 12 months or more had higher IQ scores (difference of 3·76 points, 95% CI 2·20–5·33), more years of education (0·91 years, 0·42–1·40), and higher monthly incomes (341·0 Brazilian reals, 93·8–588·3) than did those who were breastfed for less than 1 month. The results of our mediation analysis suggested that IQ was responsible for 72% of the effect on income.

**Interpretation:**

Breastfeeding is associated with improved performance in intelligence tests 30 years later, and might have an important effect in real life, by increasing educational attainment and income in adulthood.

**Funding:**

Wellcome Trust, International Development Research Center (Canada), CNPq, FAPERGS, and the Brazilian Ministry of Health.

## Introduction

Breastfeeding has clear short-term benefits for child survival through reduction of morbidity and mortality from infectious diseases.^1^ Breastfeeding also has long-term benefits. The results of a meta-analysis^2^ of 14 observational studies showed that breastfeeding was associated with an increase of 3·5 points (95% CI 1·9–5·0) on intelligence tests at childhood and adolescence. Two randomised trials^3^, ^4^ have also investigated this topic. In Belarus, intelligence quotients (IQs) at 6·5 years of age were, on average, 7·5 points higher in a group whose mothers received breastfeeding promotion than in a comparison group.^3^ In the UK, the mean IQ was higher in preterm children who were randomly allocated to receive breast milk than in those who received formula.^4^

Three observational studies^5^, ^6^, ^7^ have explored the association between breastfeeding and performance in intelligence tests in adults. In Denmark, Mortensen and colleagues^5^ noted that breastfeeding duration was positively associated with performance on the Wechsler Adult Intelligence Scale (mean age 27 years), while Richards and colleagues^6^ reported a positive association with performance in the National Adult Reading Test in participants aged 53 years in the 1946 British cohort. In the Hertfordshire cohort, participants were classified as being bottle-fed, breastfed, or mixed fed; the breastfed group had increased mean scores in the AH4 IQ test, but the association disappeared after the investigators controlled for confounding variables.^7^

Evidence from observational studies from high-income countries has been criticised because of the social patterning of breastfeeding. In particular, longer durations for mothers with high socioeconomic position than for those with low position might positively confound, and thus overestimate, the benefit of breastfeeding. Comparison of observational studies with different confounding structures has been used to improve causal inference. Brion and colleagues^8^ reported that breastfeeding was positively associated with performance in intelligence tests in the 1993 Pelotas (Brazil) and ALSPAC (UK) birth cohorts. Because breastfeeding was positively associated with family income in ALSPAC but not in Pelotas, the positive association in Brazil was probably not caused by residual confounding.

Whether or not apparently small IQ gains affect real life achievement—eg, educational attainment—is debatable. In the 1946 British Births cohort,^6^ the probability of participants obtaining advanced educational qualifications was 1·58 (95% CI 1·15–2·18) times higher in participants who had been breastfed for more than 7 months than in those who had never been breastfed. In New Zealand, breastfeeding duration was positively associated with performance in secondary school tests in students aged 18 years.^9^ However, the results of a pooled analysis^10^ of four cohort studies from low-income and middle-income countries (including data from the 23-year-follow-up visit to the 1982 Pelotas cohort) did not show consistent associations between breastfeeding duration and number of school years completed, although associations were present in two of the sites.

Because of the association between intelligence and educational attainment, the notion that breastfeeding can also increase individual income, and thus contribute to economic productivity, has been postulated.^11^, ^12^ However, our systematic review of the literature did not reveal any studies with results showing that breastfeeding was associated with income in adults.

We aimed to assess the associations between infant feeding and IQ, educational attainment, and income in participants aged 30 years in a large population-based birth cohort, in a setting where no strong social patterning of breastfeeding exists.

## Methods

### Participants

In 1982, five maternity hospitals in Pelotas, Brazil, were visited daily and all births were identified from labour ward records; 5914 neonates whose families lived in the urban area of the city were examined and their mothers were interviewed soon after delivery. The initial refusal rate was less than 1%, and the cohort has been followed up on several occasions.^13^ The study protocol is available online.

Members of the original cohort were traced in 1984 (5161 [87%] individuals) and 1986 (4979 [84%] individuals). Between June 4, 2012, and Feb 28, 2013, cohort members were invited to visit a research clinic to be interviewed and examined.

The Ethical Review Board of the Faculty of Medicine of the Federal University of Pelotas approved the study, and we obtained written informed consent from all participants.

### Procedures

Information about duration of breastfeeding and age at introduction of complementary foods was obtained in 1984, when the average age of participants was 19 months. For participants who were not interviewed in 1984, this information was obtained when they were seen in 1986 at a mean age of 42 months (SD 3·68); these 263 individuals represented 5% of the 5332 participants with infant feeding data. We defined duration of predominant breastfeeding as the age when foods other than breastmilk, teas, or water were introduced. We assessed exclusive breastfeeding but did not include it in the present analysis, because it was seldom practised at that time. We combined participants who had never been breastfed with those who were breastfed for less than 1 month because the incidence of breastfeeding was very high and evidence suggested misclassification between these two categories.^14^

We assessed intelligence using the Wechsler Adult Intelligence Scale, third version, at a mean age of 30·2 years, with the arithmetic, digit symbol, similarities, and picture completion subtests. Four psychologists who were unaware of participant feeding history administered the tests. Educational attainment was recorded as the highest grade completed successfully. In the 2012–13 visit, we asked the participants to report their income in the previous month. Information on income was gathered in Brazilian reals (R$; US$1 was worth 0·49 real in 2012).

The confounding variables measured in the perinatal study were monthly family income, maternal education, maternal smoking during pregnancy (non-smokers, 1–14 cigarettes a day, or ≥15 cigarettes a day), maternal age, maternal pre-pregnancy body-mass index (height was measured by the research team, and pre-pregnancy weight was based on information from antenatal care records or—when not available—by recall), type of delivery (caesarean or vaginal), gestational age (in full weeks, based on the date of the last menstrual period), and birthweight (from calibrated paediatric scales). Additional confounders measured during the 1984 and 1986 visits were parental education (in full years), household assets index (obtained through factor analysis and based in the ownership of household goods), and genomic ancestry. Genomic ancestry analysis was based on DNA samples that were genotyped using the Illumina Omni 2·5M array (Illumina, San Diego, CA, USA). Admixture analyses were based on 370 539 single nucleotide polymorphisms shared by samples from the HapMap Project, the Human Genome Diversity Project (HGDP), and the Pelotas cohort. The following HapMap samples were used as external panels : 266 Africans, 262 Europeans (American and Italian), 77 admixed Mexican Americans, 83 African Americans, and 93 Native Americans from the HGDP. For each individual, the proportion of European, African American, and Native American ancestry was estimated.

### Statistical analysis

We used ANOVA to compare means, and multiple linear regression to adjust the estimates for confounders. In the linear regression models, we graphically tested the normality of residuals and homoscedasticity (homogeneity of variance). We assessed multicollinearity between the explanatory variables using the variance inflation factor. We based statistical comparisons between categories on tests of heterogeneity and linear trend, and we present the one with the lower p value. We used Stata 13·0 for the analyses. We did four sets of analyses to compare breastfeeding categories in terms of arithmetic means, geometric means, median income, and to exclude participants who were unemployed and therefore had no income.

We used G-computation^15^ to estimate the direct effect of breastfeeding on income at 30 years, and the indirect effect that was mediated through IQ. In this model, base confounders included the variables listed previously. Because breastfeeding has been postulated to affect IQ in early life, we treated educational attainment as a post-confounder in the mediation analyses. We estimated the total direct and indirect effects using interactions between exposure (breastfeeding) and mediator (IQ). No statistical evidence suggested any effect modification, but we still included an interaction term in the mediation analysis.

### Role of the funding source

The funders of the study had no role in study design, data collection, data analysis, data interpretation, or writing of the report. The corresponding author had full access to all the data in the study and had final responsibility for the decision to submit for publication.

## Results

In 2012–13, we interviewed 3701 of the 5914 participants enrolled as neonates in 1982. Added to the 325 known to have died, this number represented a follow-up rate of 68%. Full information on IQ and breastfeeding was available for 3493 members. The participants interviewed in 2012–13 were slightly more likely to be female and belong to intermediate socioeconomic categories than were the original cohort (appendix). However, the magnitude of these differences was small, with the maximum difference between variable categories with the highest and lowest follow-up rates being less than 9%. Differences were also small for follow-up rates with respect to breastfeeding duration (appendix).

Table 1 shows the wide variability in maternal education. One in every five mothers breastfed for less than 1 month, and one in six did so for a year or more. Few mothers maintained predominant breastfeeding for 4 months or more (table 1). At the age of 30 years, the mean IQ of offspring was 98·0 (SD 12·6) points and the average number of years of education was 11·4 (4·1). These two variables were moderately correlated (Pearson's correlation coefficient of 0·64, p<0·0001). The distribution of monthly income was positively skewed, with a median of R$1000 (IQR 530–1890) and a mean of R$1501 (SD 1775). The results of these four analysis sets were very similar, so we report only those for arithmetic means, including all participants. Correlation coefficients were 0·39 (p<0·0001) between income and education and 0·42 (p<0·0001) between income and IQ.

**Table 1:** Characteristics of participants

		Prevalence or average value (n=3493)*
**Variables measured at birth**		
Birthweight (g)		3225 (525)*
Maternal education (years)		
	0–4	1110 (32%)
	5–8	1518 (43%)
	9–11	378 (11%)
	≥12	483 (14%)
**Variables measured during childhood**		
Duration of any breastfeeding (months)		
	<1	736 (21%)
	1–2·9	895 (26%)
	3–5·9	808 (23%)
	6–11·9	474 (14%)
	≥ 12	580 (17%)
Duration of predominant breastfeeding (months)		
	<1	894 (26%)
	1–1·9	462 (13%)
	2–2·9	687 (20%)
	3–3·9	916 (26%)
	≥4	421 (12%)
**Variables measured at 30 years of age**		
IQ		98·0 (12·6)†
Educational attainment (years)		11·4 (4·1)†
Monthly income (R$)		1000 (530–1890)‡

IQ=intelligence quotient.

* Prevalence of variables might not sum to 3493 because of missing data.

† Mean (SD).

‡ Median (IQR).

Table 2 shows the analysis with respect to the confounding variables. For breastfeeding status at 6 months, we noted a U-shaped pattern with maternal education and, to a lesser extent, family income at birth. Differences between the extreme educational attainment and income groups were less than 10%. Birthweight was directly associated with breastfeeding, but there was no difference in birthweight between the sexes. Educational attainment, IQ, and income at 30 years increased with maternal education, family income, and birthweight. Men had slightly higher IQ results than did women, but the opposite was true for educational attainment. Income was higher among men than women.

**Table 2:** Mean IQ, educational attainment, and monthly income at 30 years with respect to confounding variables

		**Breastfeeding at 6 months**	**IQ**	**Educational attainment (years)**	**Monthly income (R$)**
Maternal education at delivery, years		p<0·0001†	p<0·0001*	p<0·001*	p<0·0001*
	0–4	359/1110 (32%)	92·2 (91·5–92·9)	9·2 (9·0–9·4)	997 (931–1062)
	5–8	417/1518 (27%)	97·5 (97·0–98·1)	11·1 (11·0–11·3)	1356 (1281–1430)
	9–11	98/378 (26%)	103·2 (102·1–104·3)	13·2 (12·9–13·5)	1871 (1679–2063)
	≥12	178/483 (37%)	108·6 (107·7–109·5)	15·3 (15·1–15·6)	2846 (2614–3078)
Family income, multiple of 1982 minimum wage		p=0·03†	p<0·0001*	p<0·0001*	p<0·0001*
	≤1	209/684 (31%)	91·5 (90·7–92·4)	8·9 (8·6–9·1)	940 (855–1024)
	1·1–3	509/1722 (30%)	96·6 (96·0–97·1)	10·7 (10·6–10·9)	1255 (1191–1320)
	3·1–6	189/684 (28%)	102·0 (101·2–102·9)	13·1 (12·8–13·4)	1894 (1750–2038)
	6·1–10	69/204 (34%)	106·7 (105·2–108·2)	14·5 (14·1–14·9)	2583 (2260–2907)
	>10	72/183 (39%)	110·4 (108·9–112·0)	15·8 (15·3–16·2)	3208 (2787–3628)
Birthweight, g		p<0·0001*	p<0·0001*	p<0·0001*	p<0·0001*
	<2500	57/247 (23%)	94·4 (92·8–96·1)	10·5 (10·0–11·0)	1190 (1009–1370)
	2500–2999	227/833 (27%)	95·7 (94·8–96·5)	10·6 (10·4–10·9)	1266 (1161–1370)
	3000–3499	407/1315 (31%)	98·5 (97·8–99·1)	11·4 (11·2–11·7)	1538 (1444–1632)
	≥3500	363/1097 (33%)	99·9 (99·2–100·6)	11·9 (11·6–12·1)	1709 (1597–1821)
Sex		p=0·88†	p=0·02†	p<0·0001†	p<0·0001†
	Male	504/1677 (30%)	98·5 (97·9–99·1)	10·9 (10·7–11·1)	1917 (1826–2008)
	Female	550/1816 (30%)	97·5 (96·9–98·1)	11·7 (11·5–11·9)	1104 (1034–1173)

Data are n/N (%) or mean (95% CI) unless stated otherwise. IQ=intelligence quotient.

* Test for linear trend.

† Test for heterogeneity.

In the crude analyses, the outcome variables increased monotonically with breastfeeding duration up to 12 months, and with predominant breastfeeding duration up to 4 months (table 3). However, for both breastfeeding indicators, the longest duration groups showed slightly lower values for the outcomes than did the penultimate categories. After adjustment for confounders, the dose-response patterns for IQ and educational attainment become monotonic, although for income, the longest-duration breastfeeding groups remained slightly (but not significantly) less than the penultimate categories. For total breastfeeding, the adjusted differences between the extreme groups were 3·76 (95% CI 2·20–5·33) IQ points, 0·91 (0·42–1·40) years of education, and R$341 (93·8–588·3). Differences associated with predominant breastfeeding tended to be lower than for any breastfeeding.

**Table 3:** IQ, educational attainment, and income at 30 years, with respect to breastfeeding duration

		**IQ**		**Educational attainment (years)**		**Monthly income (R$)**	
		Mean (95% CI)	Adjusted regression β (95% CI)*	Mean (95% CI)	Adjusted regression β (95% CI)*	Mean (95% CI)	Adjusted regression β (95% CI)*
Breastfeeding duration, months		p<0·0001†	p<0·0001‡	p<0·0001†	p=0·003‡	p<0·0001‡	p<0·0001‡
	<1	96·4 (95·5–97·3)	Reference (0)	10·9 (10·6–11·2)	Reference (0)	1238 (1142–1333)	Reference (0)
	1–2·9	96·9 (96·0–97·7)	0·38 (−1·03 to 1·79)	11·0 (10·7–11·3)	0·31 (−0·13 to 0·75)	1452 (1335–1570)	222·5 (0·26 to 444·7)
	3–5·9	98·7 (97·9–99·6)	1·77 (0·35 to 3·19)	11·7 (11·4–11·9)	0·49 (0·05 to 0·94)	1584 (1458–1711)	285·1 (61·0 to 509·2)
	6–11·9	101·3 (100·1–102·5)	3·50 (1·84 to 5·16)	12·1 (11·7–12·5)	0·65 (0·12 to 1·17)	1915 (1753–2104)	485·0 (222·2 to 747·8)
	≥12	98·1 (97·0–99·1)	3·76 (2·20 to 5·33)	11·2 (10·9–11·5)	0·91 (0·42 to 1·40)	1429 (1289–1569)	341·0 (93·8 to 588·3)
Predominant breastfeeding, months		p<0·0001†	p=0·07†	p<0·0001†	p=0·02†	p<0·0001‡	p=0·12‡
	<1	96·7 (95·9–97·6)	Reference (0)	11·0 (10·7–11·2)	Reference (0)	1308 (1206–1410)	Reference (0)
	1–1·9	97·4 (96·2–98·6)	1·17 (−0·46 to 2·80)	10·8 (10·4–11·2)	0·07 (−0·44 to 0·58)	1457 (1301–1613)	241·3 (−14·2 to 496·9)
	2–2·9	98·6 (97·7–99·6)	0·70 (−0·75 to 2·15)	11·7 (11·3–12·0)	0·65 (0·20 to 1·10)	1584 (1439–1730)	207·1 (−20·0 to 434·3)
	3–3·9	99·3 (98·5–100·1)	1·38 (0·08 to 2·69)	11·7 (11·4–11·9)	0·46 (0·05 to 0·87)	1639 (1519–1760)	235·7 (30·7 to 440·6)
	≥4	97·7 (96·5–98·9)	2·29 (0·65 to 3·94)	11·4 (11·0–11·8)	0·61 (0·09 to 1·12)	1460 (1298–1622)	285·6 (26·8 to 544·3)

IQ=intelligence quotient.

* Adjusted for family income at birth, parental schooling, household score index, genomic ancestry, maternal smoking during pregnancy, maternal age, type of delivery, maternal prepregnancy body-mass index, gestational age, and birthweight.

† Test for heterogeneity.

‡ Test for linear trend.

**Figure 1:**
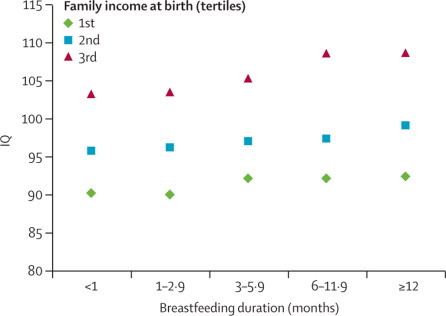
Association of mean IQ with breastfeeding duration, stratified by family income at birth Estimates are adjusted for parental education, household score index, genomic ancestry, maternal smoking during pregnancy, maternal age, type of delivery, maternal body-mass index before pregnancy, gestational age, and birthweight.

Figure 1 shows the association between IQ test performance and breastfeeding duration, according to tertiles of family income at birth. The three separate lines support a strong association between IQ and family income, as suggested in table 3. Increasing gradients seem to exist for IQ as family income at birth increased, although no evidence suggested a statistical interaction between breastfeeding and family income (p=0·94; figure 1).

**Figure 2:**
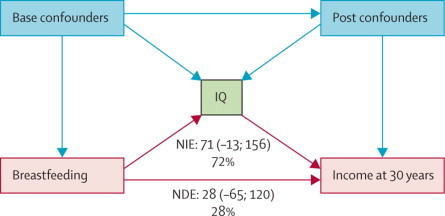
Direct acyclic graph of the effect of breastfeeding on monthly income at 30 years NIE shows that 72% of the total effect of breastfeeding on income at 30 years (99 [95% CI 6·0–192·0]) is mediated by an individual's IQ and only 28% through NDE. We adjusted estimates for base confounders—family income at birth, parental education, household score index, genomic ancestry, maternal smoking during pregnancy, and birthweight—and educational attainment as a postconfounder. NIE=natural indirect effect. NDE=natural direct effect.

Mediation analysis (figure 2) showed that adult IQ was responsible for 72% of the effect of breastfeeding on income.

## Discussion

In this population-based, prospective birth cohort, breastfeeding duration was positively and linearly associated with performance in intelligence tests, educational attainment, and income at age 30 years. The magnitude of the identified effects was important in public health terms. The difference in IQ between the most extreme groups was nearly four points, or about a third of a standard deviation; the increase of 0·9 years in education corresponds to roughly a quarter of a standard deviation, and the difference in income of R$341 was equivalent to about a third of average income.

The high follow-up rates after 30 years, the absence of differential follow-up with respect to breastfeeding duration, and the relatively similar follow-up rates for several baseline characteristics suggest that the present results are unlikely to have been affected by selection bias. All information on exposures and confounding factors were obtained in early life by trained interviewers.

The study has some potential limitations. Information on duration of breastfeeding was collected at the age of 19 months for 96% of the sample, and of 42 months for the remainder. A validation study done in a subsample of the cohort showed that 24% of mothers misclassified breastfeeding duration measured in 3-month categories, but in nearly all such cases misclassification involved neighbouring categories.^16^ The weighted kappa statistic comparing the information provided in 1984 and 1986 was 0·80, suggesting a high degree of agreement. Most scientific literature on the long-term effects of breastfeeding relies on substantially extended recall periods, with information being collected retrospectively during late childhood or adolescence.^2^ For family income data, we asked participants about the month preceding the interview. In this urban sample, seasonal employment was not common. Monthly fluctuations in income did occur, but annual income is not a common concept in Brazil, and recall might pose a problem. We also included a household wealth measure based on ownership of assets as a confounding variable to improve ascertainment of socioeconomic position. Intelligence tests were not done for parents, but we included maternal and paternal education (in a society where educational attainment varied substantially) as confounding variables; in cohort members, the correlation between IQ and years of education was 0·42.

Residual confounding by socioeconomic status should be considered in assessment of the association between breastfeeding and performance in intelligence tests, because IQ is positively related to socioeconomic position.^17^, ^18^ When the cohort started, awareness of the benefits of breastfeeding was scarce in Brazil, and no clear social patterning of breastfeeding existed for family income or parental education, unlike findings from high-income countries.^8^ Therefore, residual confounding is unlikely to explain the present findings. The potential exception is mothers who breastfed for more than 12 months, who were generally poorer, less educated, and included a larger proportion of women with higher African ancestry than did the other groups.^19^ In the present analyses, adjustment for ten potential confounders increased the estimates for IQ, educational attainment, and income in this group, leading to nearly linear associations between breastfeeding and these outcomes. When residual confounding is present, associations tend to be weakened, rather than strengthened by adjustment.

Because we neither measured home environment characteristics during childhood nor maternal-infant bonding, we were unable to explore whether the associations identified might be attributable to biological components of breastmilk itself, mother-infant bonding, or intellectual stimulation of breastfed children.^20^ However, scientific literature shows that even after controlling for home environment or stimulation, breastfed subjects have improved performance in cognitive tests,^2^ thus suggesting that breastmilk itself has a programming effect on intelligence. The findings of Lucas and colleagues' study,^4^ in which either breastmilk or formula was provided to preterm infants, suggest a direct effect of components present in milk. Additionally, the Belarus breastfeeding promotion trial^3^ did not entail any changes in the home environment, but still identified a positive effect for breastfeeding promotion. A possible biological mechanism for this effect is the presence of long-chain saturated fatty acids in breastmilk, which are essential for brain development.^21^, ^22^ Our finding that predominant breastfeeding was also positively associated with IQ at 30 years is consistent with a biological effect, suggesting that the amount of milk consumed has a role.

The scientific literature, including several observational studies and two randomised trials,^3^, ^4^ strongly suggests a causal effect of breastfeeding on performance in intelligence tests during childhood (panel).^2^ However, two questions remain. Because most available studies have tested children and adolescents, is there a long-term effect of breastfeeding on adult intelligence? This question is particularly relevant in view of fluctuations in IQ in the same individuals at different ages.^24^ Furthermore, is the magnitude of this effect important from a human capital standpoint?

Panel **Research in context**

**Systematic review**

We updated the 2013 systematic review on breastfeeding and intelligence that we reported in 2013^23^ using the same search words and methods.

**Interpretation**

Previous research, including two randomised trials, had shown clear associations between breastfeeding and child intelligence.^3^, ^4^ Some evidence suggests the presence of long-term effects on adult intelligence and school achievement.^6^, ^9^ Our study is the first to also show a positive association with adult earnings, which is largely mediated through intelligence levels.

Three studies^5^, ^6^, ^7^ assessed this association among adults. Similar to our study, Mortensen and colleagues^5^ and Richards and colleagues^6^ reported that breastfeeding duration showed dose-response associations with performance in intelligence tests, whereas Gale and colleagues^7^ did not identify an association. However, Gale and colleagues classified participants as ever or never breastfed, and might thus have underestimated the benefits of increased breastfeeding duration. Taken together with the present results, these findings suggest that the beneficial effects of breastfeeding on intelligence persist into adulthood.

Additional evidence is provided by the scientific literature on breastfeeding and educational attainment. The results of studies done in the UK^6^ and New Zealand^9^ showed positive associations, but the findings of a collaborative analysis of four cohort studies from low-income and middle-income countries were mixed.^10^ In a previous report of the 1982 Pelotas cohort at the time of Army conscription (18-year-old men), breastfeeding was associated with the number of school years completed.^19^

Because earning ability is associated with both IQ and educational attainment,^12^ breastfeeding has been postulated to have a positive economic effect on society as a whole. Economic analyses from the USA suggest that one additional IQ point increases lifetime earnings by 1·8–2·4%.^11^ A recent modelling exercise in the UK also postulated that a two point difference in IQ caused by longer breastfeeding duration would increase lifetime earnings by between £35 000 and £72 000.^25^

Nevertheless, all evidence for an effect of breastfeeding on earnings has so far been indirect; our results are the first to report a direct association. Additional evidence of a causal link is provided by our finding that the effect on income is mostly mediated through IQ. In comparisons of participants who were breastfed for 12 months or more with those breastfed for less than one month, the increase in income was roughly R$300, or 20% of the average income level. For predominant breastfeeding duration, the difference between extreme categories was of similar magnitude.

Our results suggest that breastfeeding not only improves intelligence up to adulthood, but also has an effect at both the individual and societal level, by increasing educational attainment and earning ability.

## Supplementary Material

Supplementary appendix

Supplementary audio Bernardo Lessa Horta discusses the association between breastfeeding and intelligence, educational attainment, and income at 30 years of age.
