# Supplementary material for: Association between breastfeeding and intelligence, educational attainment, and income at 30 years of age: a prospective birth cohort study from Brazil
Source: Lancet Glob Health. 2015 Apr;3(4):e199–205. doi: 10.1016/S2214-109X(15)70002-1 (PMC4365917; doi:10.1016/S2214-109X(15)70002-1)
Supplement: Supplementary appendix [file mmc1.pdf]

THE LANCET Global Health

Supplementary appendix

This appendix formed part of the original submission and has been peer reviewed. We post it as supplied by the authors.

Supplement to: Victora CG, Lessa Horta B, Loret de Mola C, et al. Association between breastfeeding and intelligence, educational attainment, and income at 30 years of age: a prospective birth cohort study from Brazil. *Lancet Glob Health* 2015; **3:** e199–205.

Appendix

Proportion of participants from the original 1982 cohort with IQ data in 2012-13, according to selected characteristics.

	Original Number of participants 1982	Participants with IQ data (N) 30 years	Proportion of participants with IQ data (%) [a]	p value
Gender				<0·001
Male	3037	1739	61·2	
Female	2876	1872	68·2	
Family income (1982 tercils minimum wages)				<0·001[b]
1st tercil	1963	1123	62·5	
2nd tercil	1979	1284	68·1	
3rd tercil	1972	1204	63·1	
Maternal education at birth (years)				0·004[b]
0 – 4	1960	1154	63·6	
5 – 8	2454	1557	67·2	
9 – 11	654	394	62·9	
≥12	839	501	60·9	
Birthweight				0.029[b]
<2500	534	259	62·3	
2500/2999	1560	971	65·9	
3000/3500	2195	1329	62·6	
>3500	1620	1051	66·8	
Smoking during pregnancy (number of cigarettes)				0·728[b]
None	3811	2349	64·8	
1 to 14	1594	963	64·7	
15 or more	509	299	62·9	
Duration of any breastfeeding in months				0·060[b]
<1	1171	736	65·5	
1 – 2·9	1405	895	65·8	
3 – 5·9	1212	808	68·9	
6 – 11·9	706	474	69·0	
≥12	838	580	70·5	

[a] The 325 participants who were known to have died were not included in the denominators.
[b] Chi-squared test for heterogeneity.
